# Cytotoxicity and Apoptosis Induced by *Chenopodium ambrosioides* L. Essential Oil in Human Normal Liver Cell Line L02 via the Endogenous Mitochondrial Pathway Rather Than the Endoplasmic Reticulum Stress

**DOI:** 10.3390/ijerph18147469

**Published:** 2021-07-13

**Authors:** Xiao-Ying Wang, Jun-Mei Hao, Qiu-Rong Ren, Hai-Ying Li, Jing-Song Wu, Xiao-Huan Zhu, Jin-Yao Chen, Ya-Nan Wang, Li-Shi Zhang

**Affiliations:** 1 West China School of Public Health, Sichuan University, Chengdu 610017, China; 2015324040013@stu.scu.edu.cn (X.-Y.W.); umbrellayy@163.com (J.-Y.C.); 2 School of Medical Technology, Suzhou Vocational Health College, Suzhou 215009, China; 3 College of Life Science, Sichuan Normal University, Chengdu 610101, China; jm198903@163.com (J.-M.H.); rqr3232@yeah.net (Q.-R.R.); rereli86@163.com (H.-Y.L.); jingsongwu3344@yeah.net (J.-S.W.); changezhuzhu@126.com (X.-H.Z.)

**Keywords:** *Chenopodium ambrosioides* L., essential oil, cytotoxicity, apoptosis, endogenous mitochondrial pathway

## Abstract

*Chenopodium ambrosioides* L. (*C. ambrosioides)* has been used as dietary condiments and as traditional medicine in South America. The oil of *Chenopodium ambrosioides* L. (*C. ambrosioides)* can be used as a natural antioxidant in food processing. It also has analgesic, sedating, and deworming effects, and can be used along with the whole plant for its medical effects: decongestion, as an insecticide, and to offer menstruation pain relief. This study was conducted to investigate the cytotoxicity and apoptosis effects of an essential oil from *C. ambrosioides* in vitro. The cytotoxicity evaluation of the essential oil from *C. ambrosioides* on human normal liver cell line L02 was assessed by 3-(4,5-dimethyl-2-thiazolyl)-2,5-diphenyl-2-H-tetrazolium bromide (MTT) assay. AO/EB dual fluorescent staining assay and Annexin V-FITC were used for apoptosis analysis. The changes in mitochondrial membrane potential (MMP) were analyzed with 5,5,6,6′-tetrachloro-1,1,3,3,-tetraethyl-imidacarbocyanine iodide (JC-1) dye under a fluorescence microscope. The level of apoptosis related protein expression was quantified by Western blot. The L02 cells were treated with the essential oil from *C. ambrosioides* at 24, 48, and 72 h, and the IC_50_ values were 65.45, 58.03, and 35.47 μg/mL, respectively. The AO/EB staining showed that viable apoptotic cells, non-viable apoptotic cells, and non-viable non-apoptotic cells appeared among the L02 cells under the fluorescence microscope. Cell cycle arrest at the S phase and cell apoptosis increased through flow cytometry in the L02 cells treated with the essential oil. MMP decreased in a concentration-dependent manner, as seen through JC-1 staining under the fluorescence microscope. In the L02 cells as shown by Western blot and qPCR, the amount of the apoptosis-related proteins and the mRNA expression levels of cytochrome C, Bax, Caspase-9, and Caspase-3 increased, Bcl-2 decreased, and Caspase-12, which is expressed in the endoplasmic reticulum, showed no obvious changes in protein amount or mRNA expression level. The essential oil form *C. ambrosioides* had a cytotoxic effect on L02 cells. It could inhibit L02 cell proliferation, arrest the cell cycle at the S phase, and induce L02 cell apoptosis through the endogenous mitochondrial pathway.

## 1. Introduction

*Chenopodium ambrosioides* L. (*C. ambrosioides*) is an annual or perennial aromatic herb that belongs to the Chenopodiaceae subfamily of plants. It is native to tropical America and is now widely distributed throughout the world [1]. It has been used for centuries by native people in South America as a dietary condiment and intraditional medicine [2]. Due to its antioxidant properties, *Chenopodium ambrosioides* L. and *Chenopodium ambrosioides* L. oil are also used as natural antioxidant additives in food processing to enhance shelf life and improve the sensory properties of food [3,4]. There are abundant secondary metabolites in the *C. ambrosioides* plant, many of which have biological activities, such as insecticidal [5,6,7], antimicrobial [8,9], antioxidant [10], etc. In recent years, research has shown that the secondary metabolites extracted from *C. ambrosioides* exhibit antitumor activity. These extract have been shown to inhibit the growth of the human lymphoma cell line Raji [11], human liver cancer cell line SMMC-7721 [12], and human chronic myeloid leukemia cell line K562 [11]. Mice that were implanted with Ehrlich ascites tumor cells as peritoneal and solid tumors were treated with *C. ambrosioides* extract, increasing their survival rate [13]. *Chenopodium* oil is a mixture of ascaridole (55.38%), *p*-cymene (16.2%), alpha-terpinene (9.7%), isoascaridole (4.3%), and limonene (3.8%) [14]. In our laboratory’s previous studies, the essential oil [15] and total flavonoids [16] from *C. ambrosioides* inhibited the growth of the human breast cancer cell line MCF-7, showing a great antitumor effect in vitro. However, many other studies have shown that the secondary metabolites extracted from *C. ambrosioides* can also have toxic effects on normal organisms. In addition, *C. ambrosioides* essential oil revealed a moderate toxicity against the peritoneal macrophages of BALB/c mice [17] and the human epidermal HaCaT cell line [18]. *C. ambrosioides* essential oil was found to be able to induce gene mutations, chromosomal breakage, and DNA damage in the mouse lymphoma cell line L5178Y [19]. Furthermore, the water extracted from *C. ambrosioides* was discovered to be able to induce chromosomal aberrations, sister chromatid exchanges, cell proliferation kinetics, and mitotic indexes in human lymphocytes [20]. However, the cytotoxicity of essential oil from *C. ambrosioides* to normal cells and its mechanism need to be further elucidated.

Based on these findings, we took human normal liver L02 cells as subjects for this study to explore the possible mechanism of cytotoxicity induced by an essential oil from *C. ambrosioides* in vitro to provide a theoretical basis for the food safety evaluation and full utilization of volatile oil resources of *C. ambrosioides* essential oil.

## 2. Materials and Methods

### 2.1. Materials

Entire *C. ambrosioides* plants were collected from vacant land in the suburbs of Chengdu, Sichuan, China and verified as *C. ambrosioides* by Professor Danwei Ma, Sichuan Normal University. The collected plants were dried in a cool place. *C. ambrosioides* essential oil was extracted through a steam distillation method, then dried with anhydrous sodium and stored at −20 °C.

### 2.2. Cell Culture

Human normal hepatocyte cell line L02 (State Key Laboratory of Biotherapy, West China Hospital, Sichuan University, Chengdu, China) was cultured in RPMI-1640 medium (Harry Bioengineering Co., Ltd., Chengdu, China) supplemented with 10% fetal calf serum (Harry Bioengineering Co., Ltd., Chengdu, China), 100 μg/mL penicillin, and 100 μg/mL streptomycin. The cells were incubated at 37 °C with 5% CO_2_ in a humidity-saturated incubator, which was used while the experiments were in the log growth phase. Experiments with the same index need to be repeated at least three times.

### 2.3. MTT Assay

The effect of the essential oil from *C. ambrosioides* on cell viability was detected by using 3-(4,5-dimethyl-2-thiazolyl)-2,5-diphenyl-2-H-tetrazolium bromide (MTT) assay. In short, L02 cells were seeded at a density of 1 × 10^4^ cells/well in a 100 μL volume of the medium in 96-well plates and allowed to rest for 24 h. The cells were then incubated in the presence of 6.25–100 μg/mL of *C. ambrosioides* essential oil for 24, 48, and 72 h. The same concentration of DMSO was used as the control vehicle in all experiments. Cell viability was examined through the incubation of the cells with 1 mg/mL of MTT for 4 h after the treatment, and then the addition of 150 μL DMSO to solubilize the formazan followed by shaking in the dark. The absorbance at 490 nm was recorded with a microplate reader (Molecular Devices, Silicon Valley, FL, USA).

### 2.4. Apoptosis Observation by Fluorescence Microscope and Analysis by Flow Cytometry

For the apoptosis experiment, the L02 cells were suspended with AO/EB staining and observed by fluorescence microscopy (Leica, Germany). Twelve microliters of the mixed liquor (50 μL cell suspension was mixed with 2 μL AO/EB dye containing 100 μg/mL AO and 100 μg/mL EB) was taken and placed on a clean glass slide to examine and make an image of the cell apoptosis. The living (VNA) cells (green), viable apoptotic (VA) cells (green), non-viable apoptotic (NVA) cells (red), and non-viable non-apoptotic (NVNA) cells (red) were clearly observed under fluorescence microscopy after AO/EB double staining.

The L02 cells were treated with the essential oil at concentrations of 12.5, 50, and 100 μg/mL for 24 h, and the negative controls were treated with DMSO. Then, the cells were stained with fluorescein isothiocyanate (FITC) and conjugated to Annexin V and PI according to the manufacturer’s instructions (BD 556547 Annexin V/FITC Apoptosis Detection Kit, BD Pharmigen, USA). The population of Annexin V−/PI−, Annexin V+/PI−, Annexin V+/PI+, and Annexin V−/PI+ cells was evaluated by anACEA NovoCyte flow cytometer (ACEA Biosciences Inc., Silicon Valley, FL, USA).

### 2.5. Mitochondrial Membrane Potential Assay

Mitochondrial membrane potential (MMP) was measured by JC-1 (5,5,6,6′-tetrachloro-1,1,3,3′-tetraethyl-imidacarbocyanine iodide) staining with fluorescence microscopy. The L02 cells (3 × 10^5^) were seeded in six-well plates treated with the essential oil at the concentrations of 12.5 and 50 μg/mL for 24 h. The negative control group was treated with DMSO, and the positive control group was treated with carbonylcyanide-p-chlorophenyl hydrazone (CCCP). At the end of the incubation, the cells were harvested by trypsinization, washed with PBS, and incubated with 5 g/mL JC-1 dye for 30 min at room temperature in the dark. Then cells were re-suspended with PBS and analyzed by fluorescence microscopy.

### 2.6. Cell Cycle Analysis by Flow Cytometry

The L02 cells were treated with the essential oil at concentrations of 12.5, 25, 50, and 100 μg/mL for 24 h, and the negative controls were treated with DMSO. The cells were collected, combined, and fixed in ice-cold 70% ethanol in PBS after treatment. Then, the cells were centrifuged, pelleted, and re-suspended in PBS containing 50 μg/mL RNase A and 100 μg/mL propidium iodide (PI). The cell cycle distribution was determined by DNA content detection with an ACEA NovoCyte flow cytometer (Beckman Coulter LH500, Beckman Coulter, Fullerton, CA, USA).

### 2.7. Western Blot Analysis

Western blot analysis was used to examine protein expression in response to various concentrations of the essential oil treatments. The L02 cells (1 × 10^6^) were seeded in six-well plates and treated with the essential oil at concentrations of 12.5, 50, and 100 μg/mL for 24 h. The negative control was treated with DMSO. The L02 cells were then washed with PBS, and lysed in a RIPA buffer in the presence of protease and a phosphatase inhibitor cocktail after treatment. Protein quantity was determined by a Bradford reagent. Then SDS-PAGE gel electrophoresis PVDF membranes were incubated with the primary antibody of cytochrome C, Bax, Bcl-2, Caspase 3, Caspase 9, and Caspase 12 overnight at 4 °C. The blots were then incubated with a secondary antibody for 2 h. The samples were then sent to Google Biotechnology Co., Ltd. (Wuhan, China) for Western blot analysis.

### 2.8. Real-Time Quantitative Polymerase Chain Reaction (qPCR) Analysis

The mRNA expression levels of cytochrome C, Bax, Bcl-2, Caspase-3, Caspase-9, and Caspase-12 in response to various concentrations of essential oil treatments were determined by real-time quantitative polymerase chain reaction (qPCR) analysis. The L02 cells (1 × 10^6^) were seeded in six-well plates and treated with the essential oil at concentrations of 12.5, 50, or 100 μg/mL for 24 h. Total RNA was extracted from the samples using a Cell Total RNA Isolation Kit (Foregene, Chengdu, China) following the manufacturer’s protocols. The RNA concentration was detected by NanoDrop TM 2000 (Thermo, Waltham, MA, USA), and the RNA quality was estimated by OD_260_/OD_280_ (1.9–2.1). The RNA concentration of each sample was quantified to the same level of 100 ng/μL and reverse-transcribed to cDNA using Iscript cDNA Synthesis Kit (Bio-Rad, USA) according to the manufacturer’s protocols. The sequences of gene-specific primers were commercially synthesized by Sangong Biotech Company (Table 1). Target genes were amplified using cDNA (1 μL), SsoFast EvaGreen Supermix (5 μL, Bio-Rad, Hercules, CA, USA), a forward primer (0.3 μL), a reverse primer (0.3 μL), and RNase-free H_2_O up to 10 μL in a CFX96 Real-Time PCR Detection System (Bio-Rad, USA) under the following conditions: enzyme activation at 98 °C for 2 min, followed by 40 cycles of denaturation at 98 °C for 5 s, and annealing/extension at 60 °C for 5 s. The melting curve at 55–60 °C for 5 s was routinely established to confirm the specificity of the primers. Data were calculated using the 2^−ΔΔCq^ method and the mRNA relative expression was normalized to an endogenous reference (β-actin).

### 2.9. Statistical Analysis

All data were representative of at least three independent experiments and were presented as mean ± standard deviation (SD). Statistical analysis was performed using SPSS version 17.0 (SPSS Inc., Chicago, IL, USA). Significant differences were analyzed by a least-significant difference (LSD) test. *p <* 0.05 was considered statistically significant.

## 3. Results

### 3.1. C. ambrosioides Essential Oil Inhibited the Proliferation of L02 Cells

**Figure 1:**
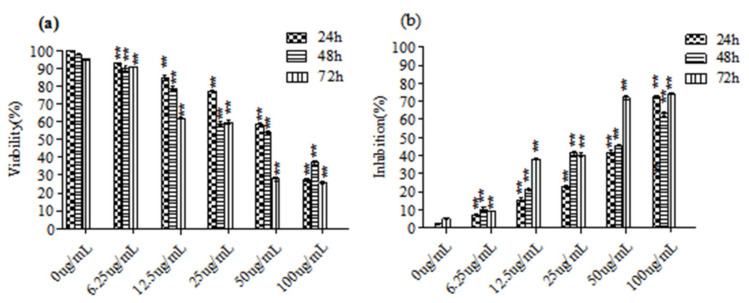
Inhibitory effect of the essential oil from *C. ambrosioides* on proliferation of the L02 cells. L02 cells were treated with the essential oil for 24, 48, and 72 h, and cell viability was determined by MTT assay. (**a**,**b**) represented the cell viability and inhibition of the L02 cells after being treated with the essential oil at different concentrations for different time respectively. The graphical plots derived from three independent experiments are shown, and the numerical values represent the means ± SEM (** *p* < 0.01, compared with the negative control).

The results of the MTT analysis of the *C. ambrosioides* essential oil’s impact on the L02 cells are shown in Figure 1. The growth of the L02 cells was inhibited significantly by the essential oil in a dose- and time-dependent manner (*p* < 0.05). The IC_50_ values of the essential oil on the L02 cells were 65.45, 58.03, and 35.47 μg/mL at 24, 48, and 72 h, respectively. Fluorouracil (80 μg/mL), as the positive control, also had an inhibitory effect on the L02 cells in a time-dependent manner. The inhibition rate was (50 ± 1)% at 24 h.

### 3.2. C. ambrosioides Essential Oil Induced Apoptosis in L02 Cells

**Figure 2:**
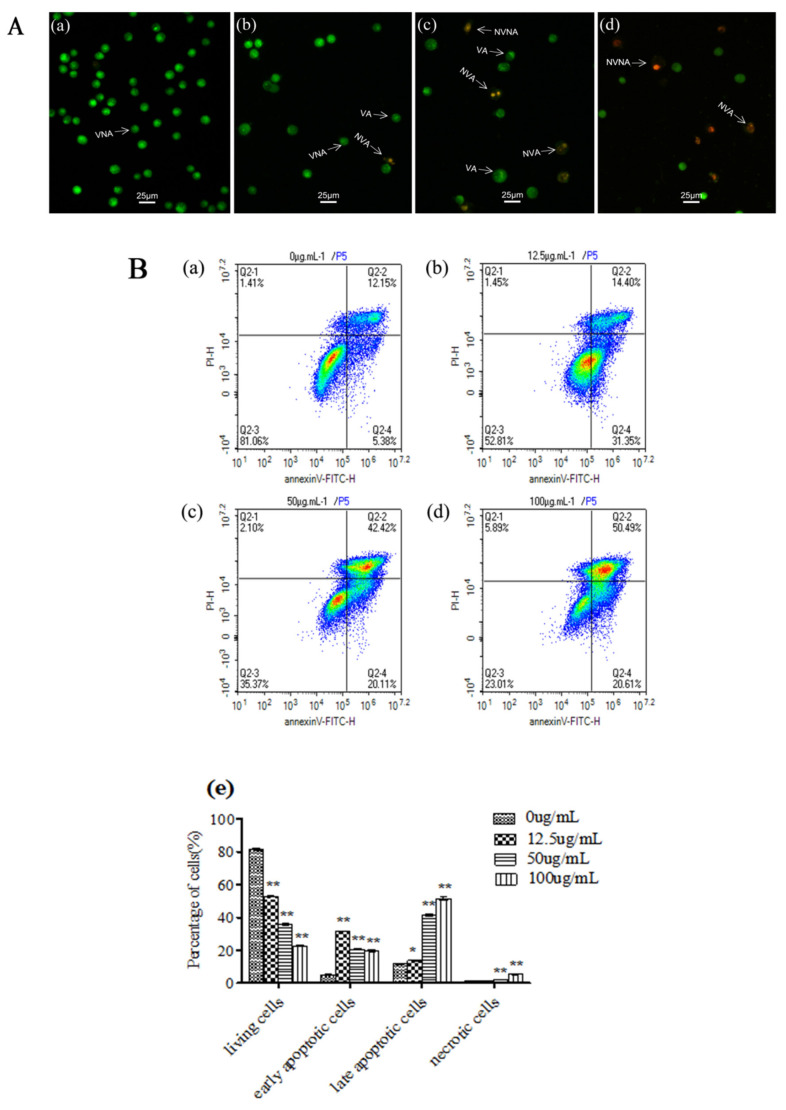

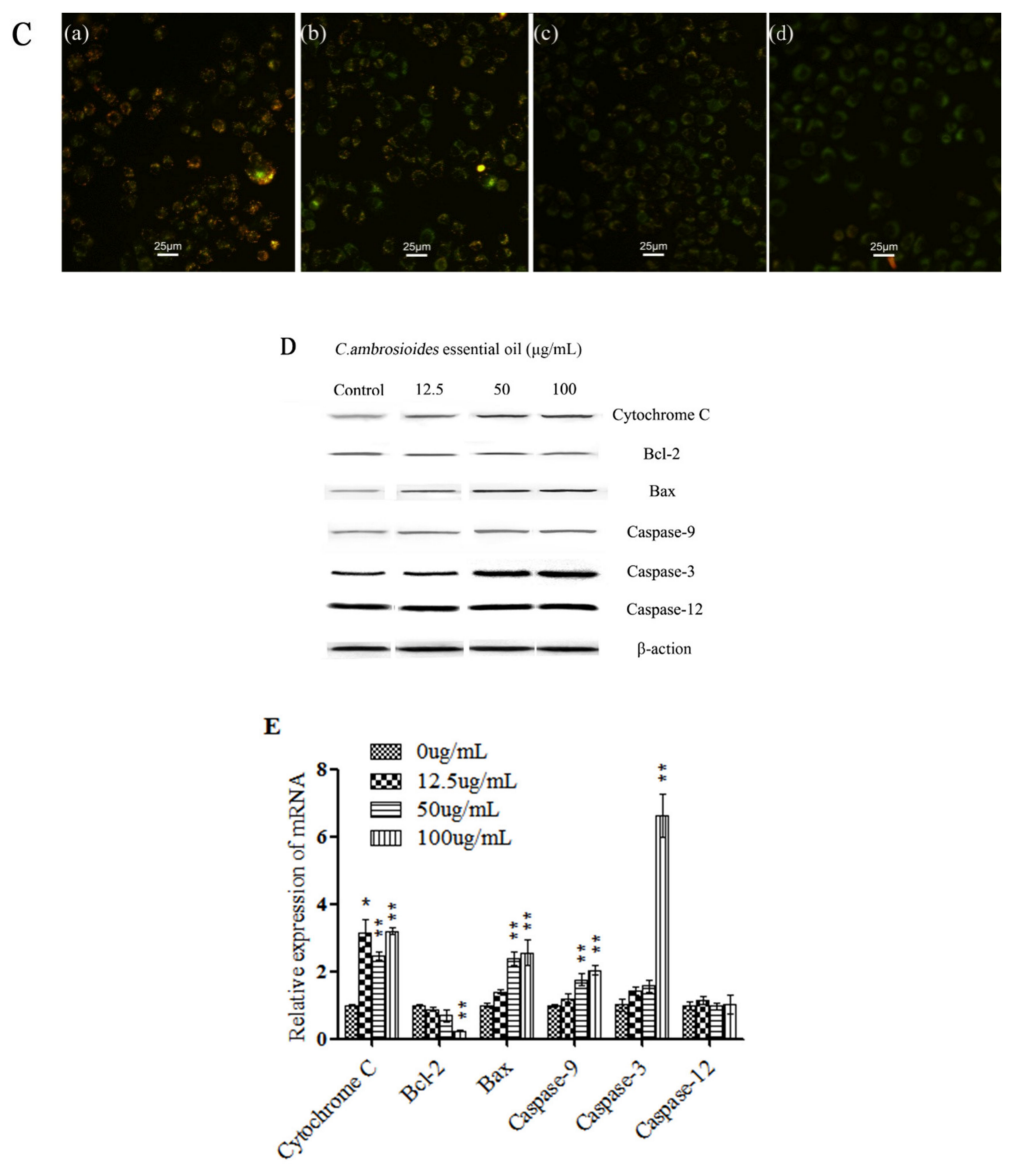
*C. ambrosioides* essential oil induced apoptosis in the L02 cells. (**A**) AO/EB double staining. (a)–(d) represented the VNA cells, VA cells, NVA cells, and NVNA cells of the L02 cells that had been treated with the essential oil for 24 h at the concentrtion of 0 µg/mL, 12.5 µg/mL, 50 µg/mL and 100 µg/mL respectively. (**B**) Cells were harvested, stained with Annexin V/PI, and data were acquired by flow cytometer. (**C**) MMP using JC-1 dyeing with fluorescence microscopy. (a)–(d) represented the the MMP of the L02 cells that had been treated for 24 h with DMSO, the essential oil at the concentrition of 12.5 µg/mL and 50 µg/mL, carbonyl cyanide-p- chlorophenyl hydrazone (CCCP) respectively. (**D**) Apoptosis related protein expression was detected by Western blot. (**E**) The mRNA expression level of apoptosis related gene was detected by qPCR. Graphical plots derived from three independent experiments, numerical values represent means ± SEM (* *p* < 0.05, ** *p* < 0.01, compared with the negative control). Note: VNA = living cells, VA = viable apoptotic cells, NVA = non-viable apoptotic cells, and NVNA = non-viable non-apoptotic cells.

For comparison with the negative control group (Figure 2Aa), the VNA cells, VA cells, NVA cells, and NVNA cells of the L02 cells that had been treated for 24 h were observed with AO/EB double staining by fluorescence microscopy (Figure 2Ab–d). The number of NVA cells increased with the increase in the essential oil concentration. Then, L02 cell apoptosis or necrosis was analyzed by staining with Annexin-V/PI after the essential oil treatment (Figure 2B). The occurrence rate of the apoptosis cells was significantly increased in a concentration-dependent manner in the treated L02 cells (*p* < 0.01). The essential oil at concentrations of 12.5 and 50 μg/mL induced VA cells among the L02 cells at a higher proportion. In addition, the essential oil could also induce L02 cell necrosis, and had a significant difference from the negative control group (*p* < 0.01).

The mitochondrion is the central organelle governed in the intrinsic apoptosis pathway (endogenous mitochondrial pathway; [21]. Therefore, in this study, the MMP of the L02 cells was measured with JC-1 dyeing under fluorescence microscopy after having been treated with the essential oil (Figure 2C). The results showed the green fluorescence (JC-1 monomers, treated as depolarized mitochondria) of the L02 cells in the CCCP positive control group (Figure 2Cd), indicating that the MMP was lower in the L02 cells. In addition, in the negative control group (Figure 2Ca), the L02 cells showed red fluorescence (JC-1 aggregates, treated as polarized mitochondria), indicating that the MMP of the L02 cells was higher. In the treatment group (Figure 2Cb,c), the red fluorescence decreased and the green fluorescence increased in a concentration-dependent manner (*p* < 0.05).

The apoptotic process is executed by a family of cysteine proteases that specifically cleave their substrates at aspartic acid residues [22] In this study, the expression of endogenous mitochondrial pathway-related proteins, including cytochrome C, Bcl-2, Bax, Caspase-9, Caspase-3, and the unique protein of the endoplasmic reticulum, Caspase-12, were detected by the Western blot method in the L02 cells after being treated with the essential oil (Figure 2D). The amount of cytochrome C, Bax, Caspase-9, and Caspase-3 increased and Bcl-2 decreased, and the unique protein Caspase-12 that expresses in the endoplasmic reticulum showed no obvious changes in the L02 cells, even with an increase in essential oil concentrations. Therefore, the essential oil from *C. ambrosioides* may induce apoptosis in L02 cells through the endogenous mitochondrial pathway.

In this study, we investigated the potential mechanism through which the essential oil from *C. ambrosioides* may induce apoptosis in L02 cells by qPCR. As shown in Figure 2E, the mRNA expression levels of cytochrome C, Bax, Caspase-3, and Caspase-9 significantly increased and Bcl-2 significantly decreased after being treated with various concentrations of the essential oil for 24 h (*p* < 0.01), and the mRNA expression levels of Caspase-12 showed no obvious changes in the L02 cells.

### 3.3. C. ambrosioides Essential Oil Induced Cell Cycle Arrest in L02 Cells

The *C. ambrosioides* essential oil induced cell cycle arrest in the L02 cells (Table 2). The population of L02 cells in the G_0/_G_1_ phase decreased from 49.43% to 31.06%, and the population of cells in the S phase increased from 35.37% to 54.08% after the cells were treated with essential oil at concentration of 100 μg/mL for 24 h. This indicates that the essential oil was able to induce L02 cell cycle arrest at S phase.

## 4. Discussion

### 4.1. Cytotoxic Effects of C. ambrosioides Essential Oil on L02 Cells

*C. ambrosioides* has been used as an anthelmintic [23], antipyretic [24], analgesic [24], antibacterial [25], and antitumor [15] drug in Brazil, Cuba, Nigeria and elsewhere. In Chinese traditional medicine, *C. ambrosioides* has been used for decongestion, to strengthen muscles and bones, as an insecticide, and to relieve pain [26]. However, the extracts of *C. ambrosioides* have certain toxic side effects, and excessive amounts can cause death in humans and rats.

In this study, the *C. ambrosioides* essential oil displayed cytotoxic activity on L02 cells. Our previous results showed that the human liver cancer cell line SMMC-7721 was treated with *C. ambrosioides* essential oil for 24 h, its IC_50_ was 26.28 μg/mL [12]. In this experiment, the IC_50_ of human normal liver cell L02 treated with the *C. ambrosioides* essential oil was 65.45 μg/mL. This indicated that the cytotoxicity of *C. ambrosioides* essential oil to human normal liver cell L02 was less than that of human liver cancer cell SMMC-7721. Electron microscopic observations indicated nuclear chromatin rupture, mitochondrial crista disruption, and different sized vacuoles forming in the cytoplasm, with some vacuoles around wrapping the cell contents and organelles. Therefore, the ultrastructural results indicated that autophagy may be involved in the process of the induction of apoptosis by *C. ambrosioides* essential oil. Meanwhile the cell cycle was arrested in S phase.

### 4.2. C. ambrosioides Essential Oil Induced L02 Cell Apoptosis through the Endogenous Mitochondrial Pathway

Apoptosis (programmed cell death) is a cellular self-destruction mechanism, and the activities of many genes influence a cell’s likelihood of activating its self-destruction program [27]. Studies have found that cytotoxic drugs induce cell apoptosis through the death factor pathway [28], the endogenous mitochondrial pathway [29], the endoplasmic reticulum stress pathway [30], autophagy [31] etc.

In this study, the number of NVA cells increased with an increase in the essential oil concentration. Mitochondria play a central role in the process of apoptosis. For L02 cells treated with the essential oil, the MMP results showed via JC-1 staining that the red fluorescence decreased and the green fluorescence increased in a concentration-dependent manner as compared to the negative control group (*p* < 0.05), indicating that the *C. ambrosioides* essential oil reduced the MMP in the L02 cells.

The MMP experiment results suggested that the *C. ambrosioides* essential oil may induce L02 cell apoptosis through the endogenous mitochondrial pathway. Subsequent experiments determined, through Western blot and qPCR, the expression of the key proteins and mRNA of genes related to the endogenous mitochondrial pathway. The representative proteins and genes are cytochrome C, Bax, Caspase-9, Caspase-3, and Bcl-2. In the endogenous pathway of apoptosis, the release of cytochrome C is critical, resulting from alterations in the permeability of the outer mitochondrial membrane [32]. Bax is a proapoptotic factor for several apoptotic signals and plays an important role in regulating the permeability of the mitochondrial outer membrane as part of the Bcl-2 protein family [33]. Caspase-9 is an initiation caspase, responsible for the precursors to the cutting effect, while Caspase-3 is an effector caspase, responsible for the structural protein in the nucleus, cytoplasm cutting and regulatory protein inactivation [34]. In our study, the expression of cytochrome C, Bax, Caspase-9, and Caspase-3, proteins, and mRNA increased and Bcl-2 protein and mRNA decreased. Caspase-12 is located in the adventitia of the endoplasmic reticulum and is a key molecule mediating ERS apoptosis. It is not activated in the death receptor or mitochondrial apoptotic pathways [35]. Our Western blot and qPCR results showed that the activity of Caspase-12 in the treatment group was almost unchanged when compared to the control group.

In this study, *C. ambrosioides* essential oil regulated the permeability of the mitochondrial extracorporeal membrane, released cytochrome C into the cytoplasm, and then induced caspases cascade activation and apoptosis, indicating that the *C. ambrosioides* essential oil induced L02 cell apoptosis, possibly through endogenous mitochondrial pathway initiation. However, the expression of the unique protein of the endoplasmic reticulum, Caspase-12, showed no obvious changes in the L02 cells, indicating that endoplasmic reticulum stress was not involved.

## 5. Conclusions

**Figure 3:**
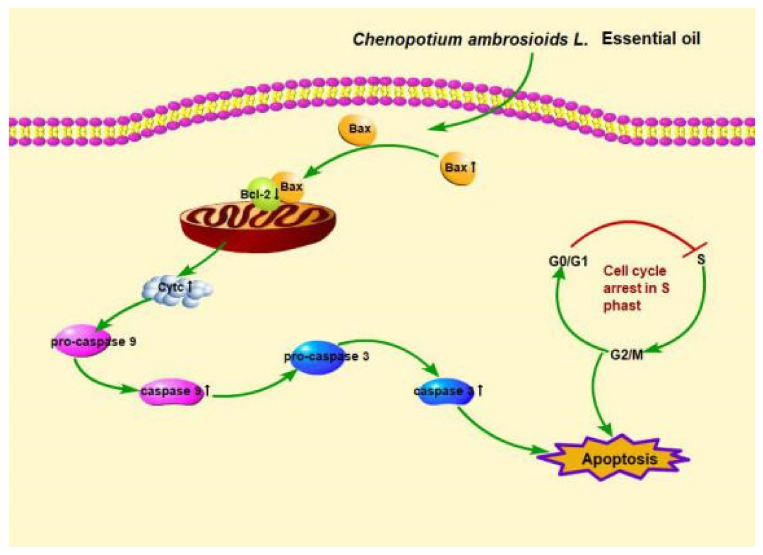
Pictorial representation depicting the mechanism of action of *C. ambrosioides* essential oil in L02 cells.

In conclusion, our study demonstrated that *C. ambrosioides* essential oil had a certain cytotoxic effect on normal human liver L02 cells, and could also induce L02 cell cycle arrest at the S phase. It induced L02 cells apoptosis through the endogenous mitochondrial pathway (Figure 3). In view of the toxic effect of the *C. ambrosioides* essential oil on normal cells, it should be used cautiously. This study provides new insights for the food safety evaluation and rational utilization of the resources of *C. ambrosioides*.

## Figures and Tables

**Table 1:** Primer sequences used for RT-PCR (5′-3′).

Gene	Sequences
Cytochrome C	Forward: CCTTTGTGGTGTTGACCAGC
	Reverse: CCATGGAGGTTTGGTCCAGT
Bcl-2	Forward: AGTGGGATGCGGGAGATGTGG
	Reverse: TAGCGGCGGGAGAAGTCGTC
Bax	Forward: GATTGCCGCCGTGGACACAG
	Reverse: GAGCACTCCCGCCACAAAGATG
Caspase-3	Forward: AGATGTCGATGCAGCAAACCTCAG
	Reverse: TGTCTCAATGCCACAGTCCAGTTC
Caspase-9	Forward: CTTCGTTTCTGCGAACTAACAGG
	Reverse: GCACCACTGGGGTAAGGTTT
Caspase-12	Forward: TACAGCTCAGGAAATGGAGACA
	Reverse: TCAATGGCTCAACACACATTCC
β-actin	Forward: GGCACTCTTCCAGCCTTCCT
	Reverse: GCACTGTGTTGGCGTACAGG

**Table 2:** Effects of *C. ambrosioides* essential oil on the cell cycle distribution of the L02 cells.

Concentrations (μg/mL)	Cell Population %		
	G_0/_G_1_	S	G_2/_M
Control	49.43 ± 2.35	35.37 ± 2.71	11.96 ± 0.55
12.5	50.96 ± 1.33	26.25 ± 5.04	11.91 ± 2.21
25	47.57 ± 3.43	34.93 ± 2.60	14.88 ± 1.26
50	42.66 ± 3.25	37.41 ± 3.42	16.51 ± 3.39
100	31.06 ± 2.01 **	54.08 ± 3.78 **	11.67 ± 3.71

Note: compared with the negative control, ** *p* < 0.01.

## Data Availability

Not applicable.
